# Supplementary figures and images for: The role of urbanisation in affecting Mytilus galloprovincialis
Source: PLoS One. 2020 May 8;15(5):e0232797. doi: 10.1371/journal.pone.0232797 (PMC7209287; doi:10.1371/journal.pone.0232797)

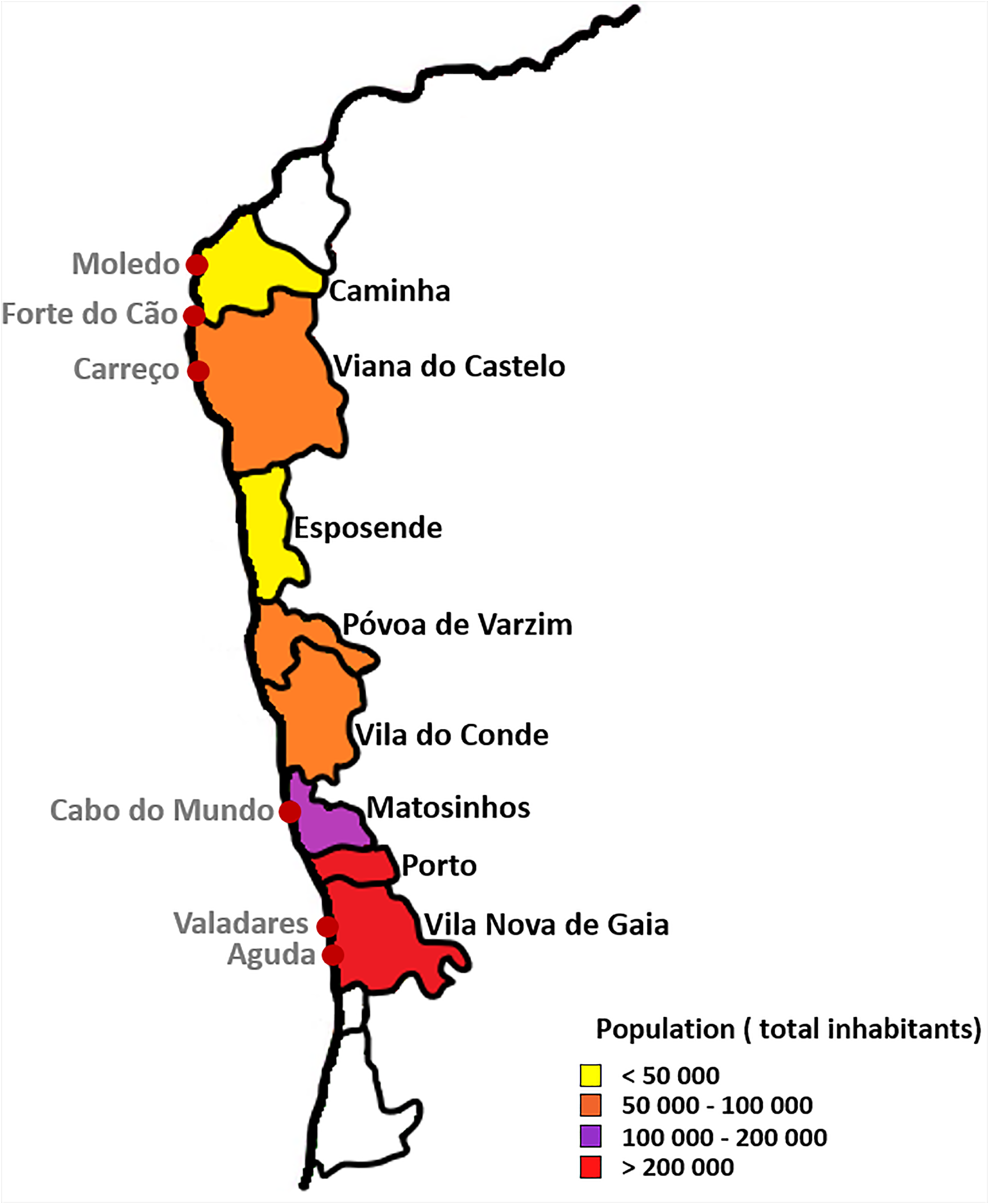

Supplement: S1 Fig — (TIF) [file pone.0232797.s001.tif]
