# Supplementary material for: The role of urbanisation in affecting Mytilus galloprovincialis
Source: PLoS One. 2020 May 8;15(5):e0232797. doi: 10.1371/journal.pone.0232797 (PMC7209287; doi:10.1371/journal.pone.0232797)
Supplement: Attachment — Submitted filename: PONE review.pdf [file pone.0232797.s003.pdf]

PONE-D-19-34175

Title

Suggest to altering to: The role of urbanisation in affecting *Mytilus galloprovincialis*

The term “attributes” is confusing without any explanation or context, especially in the title

General comments

The manuscript needs to be careful proof-reading, particularly Abstract and Introduction need extra attention. In general the findings have not been successfully discussed in terms of new ideas or the literature. Maybe the findings are not of broad interest because the authors only state a difference with density.

Abstract

I have provided an example of the changes needed to the manuscript, with an example of the Abstract:

~~Nowadays u~~Urbanisation is considered as one of the most critical and widespread threats to coastal marine ecosystems. The aim of this study was to test if urbanisation ~~shapes~~ influences the density, percentage ~~of~~ cover, ~~depth~~ thickness of clumps, condition index, and size-frequency distribution of *Mytilus galloprovincialis* in the Northern Portuguese coast. *M. galloprovincialis* ~~was selected as model because it~~ is ~~a species~~ economically and ecologically relevant. ~~A hierarchical sampling design was followed to compare m~~Mussels ~~attributes~~ were compared between urban and non-urban shores at nested spatial scales. Moreover, the relationship between mussel size and the other ~~studied attributes~~variables (i.e.??) were ~~explored~~investigated. ~~Present findings indicated that m~~Mussels ~~at~~ on urban shores showed a lower density and a higher frequency of larger individuals. A significant negative correlation between mussel length with density and with the ~~depth~~ thickness of clumps was also found. Our results indicate~~d~~ that recruitment ~~is~~ has declined ~~in~~ on urban shores and, as a result, intraspecific competition is also lower, leading to more resources being available for a ~~lesser number of~~ fewer individuals which can reach ~~higher~~ larger sizes. As mussel beds ~~harbour a~~support high biodiversity of invertebrates and provide many ecosystem services ~~to humans~~, urbanisation may ~~also~~ have indirect effects on communities associated with mussels ~~and at last to human benefits~~. ~~Therefore, knowledge about the~~Understanding the vulnerability of mussel beds to urbanisation could ~~provide~~ inform ~~for~~ management ~~not only to them but also to their associated communities and the human welfare~~.

Introduction

Sentences could be simplified, for e.g. the first sentence could read: Marine ecosystems provide food, shoreline protection against storms and floods, water quality maintenance…

Line 46 delete toughest and change menace to threats

Line 78 change unwanted to discarded

Line 79 change diminution to loss

Line 99 start new paragraph with Considering…

Line 103 other studied attributes need to be explained from the beginning as in line 192

Methods and Results

Generally the Methods and Results are written well

Line 109 write NW in full

Line 129 and throughout the manuscript the authors refer to hierarchical sampling but it is in fact a mixed model design with orthogonal and nested factors.

Table 1 change coverture to cover, put the transformation and C-test in the caption

Discussion

The Discussion requires the most work as it this stage. Rather than each paragraph starting with a finding from the literature, it should focus on the findings of the research. The Discussion could also propose a variety of explanations that may explain the observed pattern of linkages with mussels with urbanisation.
