# Supplementary material for: The role of urbanisation in affecting Mytilus galloprovincialis
Source: PLoS One. 2020 May 8;15(5):e0232797. doi: 10.1371/journal.pone.0232797 (PMC7209287; doi:10.1371/journal.pone.0232797)
Supplement: Attachment — Submitted filename: Manuscriptrev.docx [file pone.0232797.s004.docx]

**The role of urbanisation in shaping the attributes of *Mytilus galloprovincialis* populations**

Puri Veiga^1,2*^, Catarina Ramos-Oliveira^1,2^, Leandro Sampaio^1,2^, Marcos Rubal^1,2^

^1^ CIIMAR Interdisciplinary Centre of Marine and Environmental Research of the University of Porto, Novo Edifício do Terminal de Cruzeiros do Porto de Leixões, Avenida General Norton de Matos, S/N 4450-208 Matosinhos Portugal

² Department of Biology, Faculty of Sciences, University of Porto, Rua do Campo Alegre s/n, 4150-181 Porto, Portugal

* Corresponding author

Puri Veiga

E-mail: [puri.sanchez@fc.up.pt](mailto:puri.sanchez@fc.up.pt)

Short title: Urbanisation effects on mussels

**Abstract**

Nowadays urbanisation is considered as one of the most critical and widespread threat to coastal marine ecosystems. The aim of this study was to test if urbanisation shapes the density, percentage of cover, depth of clumps, condition index and size-frequency distribution of *Mytilus galloprovincialis* in the Northern Portuguese coast. *M.* *galloprovincialis* was selected as model because it is a economically and ecologically relevant species. A hierarchical sampling design was performed to compare mussel attributes between urban and non-urban shores at nested spatial scales. Moreover, the relationship between mussel size and the other attributes were explored. Results suggestedthat mussels at urban shores showed a lower density and a higher frequency of larger individuals. A significant negative correlation between mussel length with density and with the depth of clumps was also found. Our results indicated that recruitment is declined in urban shores and individuals reached higher sizes. As mussel beds harbour a high biodiversity of invertebrates and provide many ecosystem services to humans, urbanisation may also have indirect effects on communities associated with mussels and at last to human benefits. Therefore, knowledge about the vulnerability of mussel beds to urbanisation could provide support for management not only to them but also to their associated communities and the human welfare.

**Introduction**

Marine ecosystems provide many benefits for the human wellbeing such as food provision, natural shoreline protection against storms and floods, water quality, support of tourism and other cultural benefits, and maintenance of the basic global life support systems [1,2]. Despite of their importance, marine ecosystems have been strongly modified, degraded or lost as consequence of anthropogenic activities [1]. Among anthropogenic disturbances, coastal urbanisation is one the toughest, most pervasive and growing menace [3]. Human population density within 100 km of the coast is nearly three times greater than the global average [4]. Coastal areas favour the concentration of human populations because marine environment facilitates activities such as fishing, industry, tourism and transportation among other reasons [3]. Marine coastal areas urbanisation is associated to different impacts as consequence of three primary interacting drivers: exploitation of living and non-living resources, pollution pathways, both industrial and domestic, the proliferation of coastal and offshore artificial structures such as seawalls, jetties, piers, or breakwaters [4]. In this way, urbanisation is a multifaceted, heterogeneous and complex phenomenon and it is highly contextual [5]. Its ecological impacts are extreme and often irreparable such as habitat loss, spread of invasive species, disappearance of foundation species, changes in biodiversity and productivity, settlement of ruderal species, synergies in communities and proliferation of jellyfish and toxic alga [4,6,7,8,9]. Urban impacts have been deeply evaluated on terrestrial and freshwater ecosystems, showing big modification on their structure and function [4,10]. In the marine realm, most of the studies assessing the impact of urbanisation are focused on the effect of artificial structures on biodiversity [e.g. 9,11,12] and their role on the spread and settlement of non-indigenous species by comparing natural *versus* artificial habitats [e.g. 7,13]. Predictions indicate that human population living on the coast will double over the next decades, expecting that in 2025 nearly 75% of the global population will inhabit coastal areas [14]. However, understanding the effects of urbanisation on the structure and functioning of marine ecosystems has still been disregarded in the framework of conservation and management issues [15].

The Mediterranean mussel *Mytilus* *galloprovincialis* Lamarck 1819 is a widespread filter-feeding bivalve along the Atlantic rocky shores of the Iberian Peninsula, with a relevant role in intertidal food webs [16]. It is considered an ecosystem engineer species because it maintains useful habitat for other organisms, enhancing the biodiversity [17,18]. This species has long been harvested for food and bait and is now severely exploited in many European countries as Italy, Spain and Portugal [19]. Mussel harvesting has two additional adverse effects. Firstly, removal of adult mussels unavoidably eliminates juvenile mussels. Secondly, mussel beds are the preferred settlement areas for recruits. Therefore, harvesting of mussels not only depletes the adult stocks, but also diminishes recruitment and slow down recovery of mussels [20]. Mussels provide 13% of the global production of marine bivalves [21] but their farming depend on wild populations because young mussels are captured in natural systems and then deployed at culturing sites to grow-out [22]. Mussel beds are particularly vulnerable to anthropogenic disturbances [23]. For example, mussel beds in the Dutch Wadden Sea where three consecutive years of recruitment failure, intensive fishery and great rates of natural mortality removed all intertidal beds of *M. edulis* in 1990 [24]. A significant negative exponential relationship was also found between *M. galloprovincialis* recruit density and harvesting intensity, with intensities greater than 30% dramatically reducing the mussel recruitment and this pattern remained constant over 2 years [25]. Trampling and removal of *M. californianus* for bait by fisherman also diminished cover, density, biomass and size of mussels in California [26]. Moreover *M. galloprovincialis* is a species ecologically and economically relevant providing many ecosystem services as supporting (habitat for species, lifecycle maintenance, biodiversity), provisioning (food), regulating (water filtration, coastal protection) and cultural services (recreational fishing, symbolic) [27,28,29]. As mussel beds contribute to ecosystem function and the delivery of ecosystem services, understanding of their sensitivity to urbanisation may provide insights to support its assessment and management. Considering that disturbances such as harvesting or trampling are usually more intense in urban coastal areas [26,30,31], the aim of this study was to test if urbanisation shapes the density, percentage cover, depth of clumps, condition index and size-frequency distribution of *M. galloprovincialis* in the Northern Portuguese coast. Moreover, the relationship between mussel size and the other studied attributes will be explored to disentangle potential effects of intraspecific competition.

**Material and Methods**

**Study area**

The study was carried out in January 2019 at six rocky shores with different degree of urbanisation in the NW coast of Portugal. Population density was used as a proxy of urbanisation because a higher population density relates to increased threats to marine ecosystems [32]. In the study area, population is mainly concentrated near the metropolitan area of Porto, the second largest Portuguese city (S1 Fig). Three rocky shores were considered as urban: Cabo do Mundo (41.225741 N 8.717976 W), Valadares (41.089167 N 8.658374 W) and Aguda (41.04246 N 8.653254 W). Moledo (41.822605 N 8.874894 W), Forte do Cão (41.798244 N 8.88748480 W) and Carreço (41.742040 N 8.878418 W.) were considered as non-urban shores since they occur in areas near small towns (Fig 1 and S1 Fig).

**Fig 1. Location of the rocky shores.** Scale bar 50 km.

These rocky shores are characterised by granitic substrate. The tidal regime is semidiurnal, with the largest spring tides of 3.5–4.0 m. The study was done on the mid-shore, which is dominated by the mussel *Mytilus* *galloprovincialis* and the barnacles *Chthamalus* *stellatus* and *C. montagui* (Poli). Seaweeds are frequently represented by species such as *Lithophyllum* spp., *Corallina* spp., *Gelidium* spp., *Chondracanthus* *teedei* (Mertens ex Roth) Kützing and *C. acicularis* (Roth) Fredericq, *Ulva* and *Bifurcaria* *bifurcata* R. Ross [33] commonly found in tidal-pools [34].

**Sampling and sample processing**

A hierarchical sampling design was followed to compare mussel attributes between urban and non-urban shores at nested spatial scales. Two conditions (urban and non-urban) were considered. At each condition, three different rocky shores were chosen separated by 10s of km (Fig 1). Urban and non-urban sampling shores could not be interspersed because most of the population, commercial and industrial activities are concentrated near the Porto district (Fig 1 and S1 Fig). Previous studies [33,35,36,37] have indicated clear differences in pollution profiles between the shores assigned to each condition in our study. Moreover, a previous study done in norther Portugal [38] found no significant variability on rocky shore assemblages among locations separated by a similar spatial scale as urban and non-urban locations (10 s of km). Therefore, any possible confounding factor due to the spatial segregation of urban and non-urban shores was likely irrelevant compared to the specific effects under examination.

At each of the three urban and non-urban rocky shores, two sites were randomly selected (about 10 m apart). At each site, the abundance of *M. galloprovincilis* (as percentage cover) was estimated in four quadrates (50 x 50 cm). Percentage cover estimates were obtained by dividing each quadrate into 25 subquadrates of 6 x 6 cm, assigning a score from 0 (absence of mussel) to 4 (a whole sub-quadrate covered by mussel) and adding up the 25 estimates [39]. When the mussel covered less than one subquadrate, an arbitrary value of 0.5 was assigned. Additionally, four random measures of mussel clump depth were estimated for each sampled quadrate. Moreover, four quadrats (10 x 10 cm) were sampled by scraping off all mussels and samples were stored in a labelled plastic bag and frozen until further processing. In the laboratory, the number of mussels at each replicate was counted to estimate mussel density. The condition index was determined as the ratio between soft tissue dry weight and the shell dry weight at 10 replicates per quadrat (10 x 10 cm). Moreover, the shell length was measured in 20 mussels per quadrat (±0.1 mm) with a calliper, and each individual was assigned to specific size classes of shell length (Class 1: <10 mm, Class 2: 10-20 mm, Class 3: 20-30 mm, Class 4: 30-40 mm, Class 5: 40-50 mm and Class 6: >50 mm).

**Data analyses**

Analyses of variance (ANOVA) were done to test if there are differences in the density and percentage f cover of mussels among urban and non-urban shores. These analyses were based on a three-way model with condition as a fixed factor with two levels (non-urban and urban), shore as a random factor nested in condition with three levels and site randomly nested both in condition and shore with two levels and four replicates. ANOVA was also used to test for differences in the depth of clumps and condition index. These analyses were based on a four-way model, including the same factors described above for density and percentage cover plus quadrat as an additional random factor nested in condition, shore and site with four levels, and four and ten replicates for depth and condition index, respectively.

Cochran's C tests were previously done to check for homogeneity of variances, and when test was significant (p < 0.05) data were transformed to remove heterogeneity. When this was not possible, untransformed data were analysed and results were considered robust if significant at p < 0.01, to compensate for the increased probability of type I error [40]. Whenever ANOVA showed significant differences (p < 0.05), a post hoc Student-Newman-Keuls (SNK) test was done to explore differences between conditions.

To analyse differences in mussel size between urban and non-urban shores, their size-frequency was compared by means of Kolmogorov–Smirnov tests (KS).

In order to explore the relationship between mussel size and the other mussel attributes (i.e. density, percentage cover, depth of clumps and condition index), rank correlation analyses were done. Due to the non-normal distribution of the data, Spearman's rank correlation was used.

**Results**

**Effects of urbanisation on mussel attributes**

Density of mussels was significantly lower in urban shores showing a value of density nearly half of that at non-urban shores (Table 1, Fig 2A). Significant differences between conditions (i.e. urban versus non-urban) were not detected for the percentage cover, depth of clumps and condition index of mussels (Tables 1 and 2). However, these variables showed significant variability at the scale of shore and site (percentage cover), shore and quadrat (depth of clumps) and quadrat (condition index) (Tables 1 and 2; Fig 2B and 3).

**Table 1. Summary of ANOVAs for density and percentage cover of *M. galloprovincialis***.

| **Source of variation** | **df** | **Density** | | **% Cover** | |
| --- | --- | --- | --- | --- | --- |
|  |  | **MS** | ***F*** | **MS** | ***F*** |
| Condition | 1 | 3742508.52 | 7.32^*^ | 660.08 | 0.55 |
| Shore | 4 | 511616.15 | 0.96 | 1196.79 | 5.12^*^ |
| Site | 6 | 535583.85 | 2.05 | 236.67 | 3.04^*^ |
| Residual | 36 | 261227.05 |  | 76.94 |  |
| Total | 47 |  |  |  |  |
| Transformation |  | none | | none | |
| Cochran’s test |  | *C*=0.32 | ns | *C*=0.27 | ns |

ns: not significant; ^*^ p<0.05

**Fig. 2. Mussel abundance at urban and non-urban shores.** Mean values (+ SE) of density (number of mussels per 10 cm^2^) (A) and percentage cover in 50 x 50 cm (B).

**Table 2. Summary of ANOVAs for clump depth and condition index of mussels.**

| **Source of variation** | **Depth of clumps** | | | **Condition index** | | |
| --- | --- | --- | --- | --- | --- | --- |
|  | **df** | **MS** | ***F*** | **df** | **MS** | ***F*** |
| Condition | 1 | 0.56 | 0.52 | 1 | 0.008 | 1.16 |
| Shore | 4 | 1.07 | 5.94^*^ | 4 | 0.007 | 4.36 |
| Site | 6 | 0.18 | 1.74 | 6 | 0.0015 | 0.74 |
| Quadrat | 36 | 0.10 | 3.09^***^ | 36 | 0.002 | 2.10^***^ |
| Residual | 144 | 0.03 |  | 432 | 0.001 |  |
| Total | 191 |  |  | 479 |  |  |
| Transformation |  | Sqrt(X+1) | |  | none | |
| Cochran’s test |  | *C*=0.10 | ns |  | *C*=0.62 | s |

ns: not significant; s: significant; ^*^: p<0.05; ^***^ p<0.001

**Fig. 3. Depth of clumps and condition index of mussels at urban and non-urban shores.** Mean values (+SE) of depth of clumps in cm (A) and condition index (B).

In terms of size, significant differences between urban and non-urban shores were detected (KS test, Dmax = 5.9, p < 0.001). The abundance of mussels included in higher size classes was significantly larger at urban (Fig 4A) than at non-urban shores (Fig 4B).

**Fig. 4. Percentage of mussels per size classes.** At urban (A) and non-urban shores (B).

**Relationship between size and mussel attributes**

Spearman's rank correlations showed that mussel density and depth of clumps significantly decreased with mussel size, estimated as shell length (Fig 5A, 5C). However, there was no significant relationship between mussel size and percentage cover and condition index (Fig 5B, 5D).

**Fig 5. Spearman rank´s correlation between size and different attributes of mussels.** Density (A), Percentage of cover (B), Depth of clumps (C) and Condition index (D). ^*^: p < 0.05; ^**^: p < 0.01; ^ns^: Not significant.

**Discussion**

Disturbances associated with coastal urbanisation are consistently considered as the most severe and prevalent threats to global marine ecosystems [41]. Many studies estimate that population density in coastal areas will increase in the future and consequently also the coastal urbanisation [14]. The effect of urbanisation on marine resources is hardly reflected in the frame of urbanisation [4] and to fill this gap our study compared different attributes of *M. galloprovincialis*, an exploited commercial species with a relevant ecological role, between urban and non-urban conditions. The most notorious result of our study was that mussels at urban shores showed a lower density and a higher frequency of larger individuals. In freshwater habitats, different studies have also evaluated the effects of urbanisation on mussel species. In concordance with our results, Gillis et al. [42] found that urbanisation reduced the abundance of freshwater bivalves and altered the size (length) frequency distribution of the most abundant species (*Lasmigona* *costata*) causing an increase of its size. Previously Gillis [43] found that the same *L. costata*, showed a lower condition factor downstream of the urban area (more urbanised) than upstream of the cities (less urbanised). This contrasts with our results since we did not find significant differences between urban and non-urban shores for condition index. Nevertheless, for this attribute, we found significant variability at the scale of quadrat (between meters). The study by Gillis [43] did not consider nested spatial scales and this could have influenced the results.

In the marine realm, different works have proved that mussel beds are particularly vulnerable to anthropogenic disturbances. Rius and Cabral [44] found a strong decline in density of *M. galloprovincialis* at more accessible sites to humans, frequently associated with more urban areas. Carranza et al. [23] found that population of *Mytella* *strigata*, an estuarine mussel species in South America, declined in urbanised areas. *Mytilus edulis* Linnaeus 1758 showed to be sensitive to introduction or spread of non-indigenous species, habitat structure changes such as removal of substratum (extraction) and physical loss (to land or freshwater habitat), impacts typically associated with urbanisation [45]. Smith et al. [46] assessed the influence of human presence on different attributes of *M.* *californianus* populations in California. They found that percentage cover, bed thickness and biomass was lower at sites subjected to higher levels of human activities or presence. Their results [46] contrast with our observations because we did not find significant differences on these variables. This inconsistence may be due to a different responses of the two species of *Mytilus* or because California populations are submitted to a higher intensity of disturbance than our study area. Other previous studies found that intense disturbances reduced the cover of mussels, for example, Airoldi and Bulleri [47] studied the effects of urban infrastructures (breakwaters), concretely their maintenance that involves the addition of new quarried rocks over large portions of the defence structures to repair harms from storms. They found that maintenance interventions to breakwaters produced a significant decrease in the cover of *M. galloprovincialis* principally on the landward (sheltered) sides of breakwaters where maintenance was more harsh and continual. Moreover, different studies found that the structure of dominant mussel beds also differs between the landward and seaward sides of breakwaters. On the landward sides, mussels are generally larger sized and form a multi-layered matrix whilst on the seaward sides individuals are smaller sized and form mono-layered beds, which tend to be less susceptible to dislodgement by physical disturbances [13]. We did not find significant differences on the depth of mussel clumps between urban and non-urban shores. However, we found a significant negative correlation between mussel length with density and with the depth of clumps. This means that the thickest clumps are composed by a higher number of mussels (> density) but with a lower size (< shell length). Our results contrast with previous studies done in artificial structures where smaller size mussels form thinner beds [47,48]. These differences could be because in our study, sampling was always done on natural rocky substrates.

Previous studies found that the size frequency distribution of the most frequent freshwater bivalve species (i.e. *L. costata*) showed that 80% of the individuals found at the sediment surface in the most urbanised area were in the superior half of their size range [42]. These authors considered that recruitment is negatively affected by urbanisation and, consequently, individuals in urban areas show a larger size. The fishery of another bivalve, the oyster *Ostrea* *conchaphila*, in British Columbia collapsed in the 1930s and ‘40s. Its decline and failure to recover was attributed to extremely low recruitment and high juvenile mortality caused by harsh environmental conditions [49]. Previously to its collapse, Stafford [50] alerted that beds of *O. conchaphila* were thin and dominated by larger size classes, a condition interpreted as indicative of low recruits survivorship. Therefore, our results match with those found for *L costata* and *O. conchaphila* [42,49]. A potential explanation for this could be that in urban areas, recruitment is declined and, as a result, intraspecific competition is lower, leading to more resources being available for a lesser number of individuals, which can reach higher sizes, as in the urban shores of our study. The significant negative correlation between size and density also supports this. In non-urban areas, density was significantly higher but individuals showed a lower size, suggesting more recruitment and enhanced intraspecific competition. The lowest recruitment in urban areas could be the result of either low adult fecundity, low larval survival or high juvenile mortality. However, these issues should be explored in future studies.

Philippart et al. [51] indicate that the highest occurrence probability of mussel larvae in the Iberian Peninsula was in late winter (end of February) and early fall (end of September). Their results also showed that the presence of mussel larvae is not determined by small-scale (local) variation in environmental conditions, but by environmental conditions that operate at larger (100s and 1000s of km) spatial scales. Therefore, variation in the larva supply does not appear to be responsible for different patterns found in our study between urban and non-urban shores. Considering the fact that in invertebrates the reproductive potential (gonad volume) increases exponentially with size, being larger animals more fecund than small individuals [46,52], and that we found a higher frequency of larger sized mussels in urban areas, the reproductive capacity should not be the most plausible explanation for our results. However, urbanisation could alter the reproductive process and the importance of fertilisation success should also be considered. At high population density, increased fertilization success can compensate for decreased gamete production. Therefore, small individuals at high population density, as in our non-urban shores, may have similar per capita zygote production as large individuals at low density populations, as in our urban shores [53], providing a reasonable explanation for our results.

Other impacts commonly associated with urban intertidal areas are harvesting and trampling [e.g. 30,31]. Different studies have shown that harvesting causes a reduction in mussel abundance and a decrease in the mussel size [30,44] so harvesting probably is not the main driver responsible for patterns found in our study at least for the larger size mussels observed in urban shores. Regarding trampling, this affects mussels directly, by removing all or part of an individual through crushing and dislodgement, or by weakening attachment strength. Thus, it increases the risk of displacement through storms or waves, the susceptibility to predation and the vulnerability of young individuals to the boarder effect [54,55]. However, Smith and Murray [26] found a shift towards higher frequencies of smaller individuals as effect of trampling. Therefore, trampling seems not to be a likely driver to explain our results of higher mussel size in urban shores submitted to more human visits (more trampling).

Puccinelli et al. [56] assed if the proximity to urban centres influenced the dietary regime of marine benthic filter feeders. They found that mussels from urbanised sites had fatty acid signatures enriched with a higher proportion of polyunsaturated fatty acids (PUFA), indicative of exposure to high food availability and quality. As urbanisation promotes nutrient input into the sea, it may also enhance primary production and thus the amount of PUFA in phytoplankton. Puccinelli et al. [56] conclude that urbanisation increases the availability of PUFA for benthic filter feeders and therefore nourishing and suitable food accessibility. This could explain why mussels in urban areas reach a higher size. Similar effects were found for organic matter supply from fish farms. Mussels close to those farms, below direct organic release, reached higher sizes than mussels distant from the farms [57,58]. Organic waste from fish farms that disperses in the water column might be a food resource for mussels, which as filter feeders are essentially generalist consumers of particulate organic matter [59] and exploit organic matter from various sources (autochthonous, terrigenous natural allochthonous or anthropogenic) according to its availability. Thus an organic matter or nutrient increase associated to punctual wastewater seepages in urban areas, dispersed by the strong wave currents, could also explain the higher mussel size observed at urban shores in our study.

Our study used a sampling design including different nested spatial scales from meters to 10s of kilometres and it was done in winter. Previous studies done in spring found also that mussels, in urban areas of the Northern Portuguese coast, reported higher mussel size than at non-urban shores [60]. This means that this pattern seems to be consistent during winter and spring but future studies should explore its consistence along the time.

We considered for our study two variables that measure the abundance of mussels, percentage of cover and density. However, we only found significant differences between urban and non-urban conditions for density whereas percentage of cover showed significant variability at the scales of shore and site. Moreover, the correlation between both variables was not significant. This means that percentage of cover is not a good proxy to estimate the mussel abundance and provides different information (i.e. habitat occupancy). Percent of cover indicates the amount of area occupied by *M. galloprovincialis* whereas density indicates the number of individuals found in a unit area (i.e. 10 cm^2^). Most of the studies done in rocky intertidal use percentage of cover that has the strong advantage of being a non-destructive method, however, our results showed that density seems to be a better descriptor to evaluate the effects of anthropogenic stressors, such as urbanisation. This result should be considered when designing future monitoring programs.

Our results therefore show that coastal urbanisation seems to have an effect on mussel attributes in terms of density and size. Moreover, as mussel beds are ecosystem-engineers that harbour a high number of individuals and species and provide many ecosystem services to mankind [e.g. 28,29], urbanisation can also have indirect effects on communities associated with mussels and ultimately to human welfare. In this way, evaluating the vulnerability of mussel beds may support assessment and management concerning not only them but also their associated communities and the human welfare.

**Acknowledgements**: This research was developed under the Project No. 30181, co-financed by COMPETE 2020, Portugal 2020 and the European Union through the ERDF, and by FCT through national funds.

**Key words**: Urbanisation, *Mytilus galloprovincialis*, Iberian Peninsula, rocky shores, nested sampling design

**References**

1. UNEP. Marine and coastal ecosystems and human wellbeing: A synthesis report based on the findings of the Millennium Ecosystem Assessment. UNEP; 2006.

2. Selig ER, Hole DG, Allison EH, Arkema KK, McKinnon MC, Chu J, et al. Mapping global human dependence on marine ecosystems. Conserv Lett. 2019; 12:e12617. <https://doi.org/10.1111/conl.12617>

3. Barragan JM, de Andrés M. Analysis and trends of the world's coastal cities and agglomerations. Ocean Coast Manage. 2015; 114:11–20. <http://dx.doi.org/10.1016/j.ocecoaman.2015.06.004>

4. Todd PA, Heery EC, Loke LHL, Thurstan RH, Kotze DJ, Swan C. Towards an urban marine ecology: characterizing the drivers, patterns and processes of marine ecosystems in coastal cities. Oikos. 2019; 128:1215–1242. <http://dx.doi.org/10.1111/oik.05946>

5. Celliers L, Ntombela C. Urbanisation, Coastal Development and Vulnerability, and Catchments. In: Paula J editor. The Regional State of the Coast Report: Western Indian Ocean. United Nations Environment Programme/Nairobi Convention Secretariat; 2015, pp. 377–395. <http://dx.doi.org/10.18356/cd2f1dd6-en>

6. Duarte CM, Pitt KA, Lucas CH, Purcel1 JE, Uye S, Robinson K, et al. Is global ocean sprawl a cause of jellyfish blooms? Front Ecol Environ. 2013; 11(2):91–97. <http://dx.doi.org/10.1890/1102462013>

7. Dafforn KA, Glasby TM, Airoldi L, Rivero NK, Mayer-Pinto M, Johnston EL. Marine urbanization: an ecological framework for designing multifunctional artificial structures. Front Ecol Environ. 2015; 13(2):82–90. <http://dx.doi.org/10.1890/140050>

8. Bugnot AB, Hose GC, Walsh CJ, Floerl O, French K, Katherine A. Dafforn KA, et al. Urban impacts across realms: Making the case for inter-realm monitoring and management. Sci Total Environ. 2019; 648:711–719. <https://doi.org/10.1016/j.scitotenv.2018.08.134>

9. Malerba ME, White CR, Dustin J Marshall DJ. The outsized trophic footprint of marine urbanization. Front Ecol Environ. 2019; 17(7):400–406. <https://doi.org/10.1002/fee.2074>

10. Mayer-Pinto M, Cole VJ, Johnston EL, Bugnot A, Hurst H, Airoldi L, et al. Functional and structural responses to marine urbanisation. Environ Res Lett. 2018; 13:014009. <https://doi.org/10.1088/1748-9326/aa98a5>

11. Glasby TM. Differences between subtidal epibiota on Pier Pilings and Rocky Reefs at Marinas in Sydney, Australia. Estuar Coast Shelf S. 1999; 48:281–290. <https://doi.org/10.1006/ecss.1998.0417>

12. Bulleri F, Chapman MG, Underwood AJ. Intertidal assemblages on seawalls and vertical rocky shores in Sydney Harbour, Australia. Austral Ecol. 2005; 30:655–667. <https://doi.org/10.1111/j.1442-9993.2005.01507.x>

13. Bulleri F, Airoldi L. Artificial marine structures facilitate the spread of a nonindigenous green alga, *Codium* *fragile* ssp. *tomentosoides*, in the north Adriatic Sea. J Appl Ecol. 2005; 42:1063–1072. <https://doi.org/10.1111/j.1365-2664.2005.01096.x>

14. Airoldi L, Beck MW. Loss, status and trends for coastal marine habitats of Europe Oceanogr Mar Biol Annu Rev. 2007; 45:345–405.

15. Bulleri F. Is it time for urban ecology to include the marine realm? Trends Ecol Evol. 2006; 21(12):658–659. <https://doi.org/10.1016/j.tree.2006.09.004>

16. Vinagre C, Mendoça V, Narciso L, Madeira C. Food web of the intertidal rocky shore of the west Portuguese coast - Determined by stable isotope analysis. Mar Environ Res. 2015; 110:53–60. <https://doi.org/10.1016/j.marenvres.2015.07.016>

17. Gestoso I, Arenas F, Rubal M, Veiga P, Peña M, Olabarria C. Shifts from native to non-indigenous mussels: enhanced habitat complexity and its effects on faunal assemblages. Mar Environ Res. 2013; 90:85–95. <https://doi.org/10.1016/j.marenvres.2013.05.015>

18. Arribas LP, Donnarumma L, Palomo MG, Scrosati A. Intertidal mussels as ecosystem engineers: their associated invertebrate biodiversity under contrasting wave exposures. Mar Biodivers, 2014; 44:203–211. <https://doi.org/10.1007/s12526-014-0201-z>

19. Bertocci I, Dominguez R, Freitas C, Sousa-Pinto I. Patterns of variation of intertidal species of commercial interest in the Parque Litoral Norte (north Portugal) MPA: Comparison with three reference shores. Mar Environ Res. 2012;77 60–70. <https://doi.org/10.1016/j.marenvres.2012.02.003>

20. Harris J, Branch G, Sibiya C, Bill C. The Sokhulu subsistence mussel-harvesting project: co-management in action. In: Hauck M, Sowman M, editors. Waves of Change: Coastal and Fisheries Co-management in Southern Africa. UCT Press Cape Town; 2003. pp. 61–98.

21. Wijsman JWM, Troost K, Fang J, Roncarati A. Global Production of Marine Bivalves. Trends and Challenges. In: Goods and Services of Marine Bivalves. Edited by Smaal A., Ferreira J., Grant J., Petersen J., Strand Ø. Springer, Cham; 2019. <https://doi.org/10.1007/978-3-319-96776-9_2>

22. Piñeiro-Corbeira C, Barrientos S, Olmedo M, Cremades J, Barreiro R. By-catch in no-fed aquaculture: exploiting mussel seed persistently and extensively disturbs the accompanying assemblage. ICES J Mar Sci. 2018; 75(6):2213–2223. <https://doi.org/10.1093/icesjms/fsy107>

23. Carranza A, Defeo O, Beck M, Castilla JC. Linking fisheries management and conservation in bioengineering species: the case of South American mussels (Mytilidae). Rev Fish Biol Fisheries. 2009; 19:349–366. <https://doi.org/10.1007/s11160-009-9108-3>

24. Beukema JJ, Cadée GC. Consequences of the Sudden Removal of nearly all mussels and cockles from the Dutch Wadden Sea. Mar Ecol. 1996;17 (1-3): 279-289. <https://doi.org/10.1111/j.1439-0485.1996.tb00508.x>

25. Robinson TB, Branch GM, Griffiths CL, Govender A. Effects of experimental harvesting on recruitment of an alien mussel *Mytilus* *galloprovincialis*. J Exp Mar Biol Ecol. 2007; 345:1–11. <https://doi.org/10.1016/j.jembe.2006.12.019>

26. Smith JR, Murray SN. The effects of experimental bait collection and trampling on a *Mytilus* *californianus* mussel bed in southern California. Mar Biol. 2005; 147:699–706. <https://doi.org/10.1007/s00227-005-1619-4>

27. Beaumont NJ, Austen MC, Atkins JP, Burdon D, Degraer S, Dentinho TP, et al. Identification, definition and quantification of goods and services provided by marine biodiversity: implications for the ecosystem approach. Mar Pollut Bull. 2007;54:253–265. <http://dx.doi.org/10.1016/j.marpolbul.2006.12.003>

28. Montes MO, Hanna SK, Lenihan HS, Keller AA. Uptake, accumulation, and biotransformation of metal oxide nanoparticles by a marine suspension-feeder. J hazard mater. 2012; 225:139–145. <https://doi.org/10.1016/j.jhazmat.2012.05.009>

29. Gundersen H, Bryan T, Chen W, Moy FE, Sandman AN, Sundblad G, et al. Ecosystem Services in the coastal zone of the Nordic Countries. Nordic Council of Ministers; 2017. <http://dx.doi.org/10.6027/TN2016-552>

30. Rius M, Kaehler S, McQuaid CD. The relationship between human exploitation pressure and condition of mussel populations along the south coast of South Africa. S Afr J Sci. 2006; 102:130–136.

31. McPhee DP. Urban Recreational Fisheries in the Australian Coastal Zone: The Sustainability Challenge. Sustainability. 2017; 9:422; <http://dx.doi.org/10.3390/su9030422>

32. Creel L. Ripple effects: population and coastal regions. Making the Link Population Reference Bureau. 2003; 8 pp.

33. Rubal M, Veiga P, Reis PA, Bertocci I, Sousa-Pinto I. Effects of subtle pollution at different levels of biological organisation on species-rich assemblages. Environ Pollut. 2014; 191:101–110. <http://dx.doi.org/10.1016/j.envpol.2014.04.019>

34. Rubal M, Veiga P, Vieira R, Sousa-Pinto I. Seasonal patterns of tidepool macroalgal assemblages in the North of Portugal. Consistence between species and functional group approaches. J Sea Res. 2011; 66:187–194. <http://dx.doi.org/10.1016/j.seares.2011.07.003>

35. Reis PA, Salgado MA, Vasconcelos V. *Chthamalus* *montagui* as biomonitor of metal contamination in the northwest coast of Portugal. Environ Monit Assess. 2012; 184:5421–5437. <http://dx.doi.org/10.1007/s10661-011-2350-x>

36. Reis P.A., Salgado, M.A., Vasconcelos, V., 2013. Seasonal variation of metal contamination in the barnacles *Pollicipes* *pollicipes* in northwest coast of Portugal show clear correlation with levels in the surrounding water. Mar Pollut Bull. 2013; 70:155–161. <http://dx.doi.org/10.1016/j.marpolbul.2013.02.027>

37. Reis PA, Cassiano J, Veiga P, Rubal M, Sousa-Pinto I. *Fucus* *spiralis* as monitoring tool of metal contamination in the northwest coast of Portugal under the European Water Framework Directives. Environ Monit Assess. 2014; 186:5447–5460. [http://dx.doi.org/10.1007/s10661-014-3794-6.Rubal et al 2014](http://dx.doi.org/10.1007/s10661-014-3794-6.Rubal%20et%20al%202014)

38. Veiga P, Rubal M, Vieira R, Arenas F, Sousa-Pinto I. Spatial variability in intertidal macroalgal assemblages on the North Portuguese coast. Consistence between species and functional groups approaches. Helgol Mar Res. 2013; 67:191–201. <http://dx.doi.org/10.1007/s10152-012-0315-2>.

39. Dethier MN, Graham ES, Cohen S, Tear LM. Visual versus random-point percent cover estimations: “objective” is not always better. Mar Ecol Prog Ser. 1993; 96:93–100.

40. Underwood AJ. Experiments in Ecology: Their Logical Design and Interpretation Using Analysis of Variances. Cambridge: Cambridge University Press; 1997.

41. Stuart-Smith RD, Edgar GJ, Stuart-Smith JF, Barrett NS, Fowles AE, Nicole A. et al. Loss of native rocky reef biodiversity in Australian metropolitan embayments. Mar Pollut Bull. 2015; 95:324–332. <http://dx.doi.org/10.1016/j.marpolbul.2015.03.023>

42. Gillis PL, McInnis R, Salerno J, de Solla SR, Servos MR, Leonard EM. Freshwater mussels in an urban watershed: Impacts of anthropogenic inputs and habitat alterations on populations. Sci Total Environ. 2017; 574:671–679. <http://dx.doi.org/10.1016/j.scitotenv.2016.09.110>

43. Gillis PL. Cumulative impacts of urban runoff and municipal wastewater effluents on wild freshwater mussels (*Lasmigona* *costata*). Sci Total Environ. 2012; 431:348–356. <http://dx.doi.org/10.1016/j.scitotenv.2012.05.061>

44. Rius M, Cabral HE. Human harvesting of *Mytilus* *galloprovincialis* Lamarck, 1819, on the central coast of Portugal. Sci. Mar. 2004; 68:545-551.

45. Mainwaring K, Tillin H, Tyler-Walters H. Assessing the sensitivity of blue mussel beds to pressures associated with human activities. Peterborough: Joint Nature Conservation Committee, JNCC Report No. 506; 2014.

46. Smith JR, Fong P, Ambrose RF. The impacts of human visitation on mussel bed communities along the California Coast: Are regulatory marine reserves effective in protecting these communities? Environ Manage. 2008; 41:599–612. <http://dx.doi.org/10.1007/s00267-007-9066-2>

47. Airoldi L, Bulleri F. Anthropogenic Disturbance Can Determine the Magnitude of Opportunistic Species Responses on Marine Urban Infrastructures. PLoS ONE. 2010; 6(8):e22985. <http://dx.doi.org/10.1371/journal.pone.0022985>

48. Airoldi L, Bacchiocchi F, Cagliola C, Bulleri F, Abbiati M. Impact of recreational harvesting on assemblages in artificial rocky habitats. Mar Ecol Prog Ser. 2005; 299:55–66. <http://dx.doi.org/10.3354/meps299055>

49. Beck MW, Brumbaugh RD, Airoldi L, Carranza A, Coen LD, Crawford C, et al. Shellfish Reefs at Risk: A Global Analysis of Problems and Solutions. The Nature Conservancy, Arlington VA. 52 pp; 2009.

50. Stafford J. The native oyster of British Columbia. Report of the Commissioner of Fisheries; 1918.

51. Philippart CJM, Amaral A, Asmus R, van Bleijswijk J, Bremner J, Buchholz F. Spatial synchronies in the seasonal occurrence of larvae of oysters (*Crassostrea* *gigas*) and mussels (*Mytilus* *edulis*/*galloprovincialis*) in European coastal waters. Estuar Coast Shelf S. 2012; 108:52–63. <http://dx.doi.org/10.1016/j.ecss.2012.05.014>

52. Underwood AJ. Exploitation of species on the rocky coast of New South Wales (Australia) and options for its management. Ocean Coast Manage. 1993; 20:41–62. <https://doi.org/10.1016/0964-5691(93)90012-N>

53. Levitan DR. Influence of body size and population density on fertilization success and reproductive output in a free-spawning invertebrate. Biol Bull. 1991; 181:261–268. <http://www.jstor.org/stable/1542097>

54. Brosnan DM, Crumrine LL. Effects of human trampling on marine rocky shore communities. J Exp Mar Biol Ecol 1994; 177:79–97. <https://doi.org/10.1016/0022-0981(94)90145-7>

55. Ferreira AM, Seixas S, Rijo A, Faria S, Fialho V. Impact of harvest by humans on mussel populations around Easter. Journal of Integrated Coastal Zone Management 2013; 13:145–155. <http://dx.doi.org/10.5894/rgci383>

56. Puccinelli E, Noyon M, McQuaid CD. Does proximity to urban centres affect the dietary regime of marine benthic filter feeders? Estuar Coast Shelf S. 2016; 169:147–157. <http://dx.doi.org/10.1016/j.ecss.2015.12.017>

57. Sarà G, Zenone A, Tomasello A. Growth of *Mytilus* *galloprovincialis* (mollusca, bivalvia) close to fish farms: a case of integrated multi-trophic aquaculture within the Tyrrhenian Sea. Hydrobiologia 2009; 636:129–136. <http://dx.doi.org/10.1007/s10750-009-9942-2>

58. Lander TR, Robinson SMC, MacDonald BA, Martin JD. Enhanced Growth Rates and Condition Index of Blue Mussels (*Mytilus* *edulis*) Held at Integrated Multitrophic Aquaculture Sites in the Bay of Fundy. J Shellfish Res. 2012; 31(4):997–1007. <http://dx.doi.org/10.2983/035.031.0412>

59. Dame RF. Ecology of Marine Bivalves: An Ecosystem Approach. CRC Press, Boca Raton, FL, USA; 1996

60. Torres AC, Rubal M, Sousa-Pinto I, Costa-Garcia R, Veiga P. Effects of urbanization on attributes and associated fauna of mussel beds. Front Mar Sci Conference Abstract: IMMR'18 | International Meeting on Marine Research 2018; <http://dx.doi.org/10.3389/conf.FMARS.2018.06.00014>

**Supporting information**

**S1 Fig. Distribution of human population along the study area**.

**S2 Table. Full data.**
